# Establishing Proposition 65 Safe Harbor Levels for Estragole and Methyleugenol in Cosmetics: A Mechanistic Read-Across from Safrole

**DOI:** 10.3390/toxics14090782

**Published:** 2026-09-04

**Authors:** Gabriela de Oliveira Prado Corrêa, Tugstênio Souza, Eduarda Naomi Nakano Conde, Andréia Ávila Soares Oliveira, Natália Albuquerque Vita, Carolina Motter Catarino, Andrezza di Pietro Micali Canavez

**Affiliations:** Safety Assessment Management, Grupo Boticário, R. Alfredo Pinto, 2258, Parque da Fonte, São José dos Pinhais 83050-320, PR, Brazil; tugstenio.souza@grupoboticario.com.br (T.S.); eduarda.conde@grupoboticario.com.br (E.N.N.C.); andreia.avila@grupoboticario.com.br (A.Á.S.O.); nataliav@grupoboticario.com.br (N.A.V.); carolina.catarino@grupoboticario.com.br (C.M.C.); andrezza@grupoboticario.com.br (A.d.P.M.C.)

**Keywords:** Proposition 65, read-across, estragole, methyleugenol, safrole, fragrance ingredients, cosmetic safety, New Approach Methodologies (NAMs)

## Abstract

California’s Proposition 65 requires warning labels for chemicals associated with carcinogenicity or reproductive toxicity, affecting global cosmetic and fragrance formulations. While safrole has an established safe harbor level (No Significant Risk Level, NSRL) of 3 µg/day, its structural analogues, estragole and methyleugenol, lack specific safe harbor thresholds. To address this data gap using non-animal methodologies, this study evaluated a Next-Generation Risk Assessment (NGRA) workflow combining read-across and *in silico* modeling. Structural, physicochemical, and skin-permeation profiling supported category homogeneity (Tanimoto *Tc* ≥ 0.82 and comparable log *Kp* values) in accordance with OECD and ECHA guidelines. Multi-platform QSAR consensus (ToxTree, Danish QSAR, and VEGA QSAR) indicated that while parent phenylpropenes lack direct bacterial mutagenicity, they share a hepatic bioactivation pathway leading to mammalian clastogenicity and carcinogenicity predictions. Molecular docking with human Cytochrome P450 1A2 (CYP1A2; PDB: 2H14) identified safrole as the category’s conservative toxicological benchmark, exhibiting the highest binding affinity (−8.026 kcal/mol) and closest proximity to the catalytic HEME iron. Consequently, applying safrole’s 3 µg/day NSRL to estragole and methyleugenol provides a quantitative threshold to evaluate consumer exposure in cosmetic products, ensuring public health protection and regulatory compliance.

## 1. Introduction

The growing concern over human exposure to potentially toxic chemicals has accelerated the development of increasingly stringent international environmental and public health regulations. Within this context, regulatory toxicology plays a fundamental role in identifying, characterizing, and managing the risks associated with chemical agents across global supply chains, directly affecting diverse sectors including food, cosmetics, and consumer goods [1]. Advanced toxicological safety assessments based on robust scientific evidence have become essential to support corporate decisions and ensure public transparency [2]. Among these international frameworks, California’s Safe Drinking Water and Toxic Enforcement Act of 1986, widely recognized as Proposition 65, has consolidated itself as one of the most influential legislations [3]. Scientifically administered by the Office of Environmental Health Hazard Assessment (OEHHA), Proposition 65 aims to protect the population against significant exposures to chemicals known to cause cancer, birth defects, or reproductive toxicity, requiring clear warnings when safety limits are breached [4,5]. The official list currently contains 879 chemical entries (as of August 2026), ranging from synthetic solvents and pesticides to naturally occurring compounds [6]. Although Proposition 65 is an individual U.S. state statute, its profound economic impacts cross international borders due to the sheer size of the Californian market, prompting global manufacturers to integrate systematic toxicological evaluations into product development and raw-material selection [1,3,7].

The presence of a substance on the Proposition 65 list does not mandate an immediate commercial ban, as the statute is fundamentally grounded in evaluating chronic, lifetime cumulative exposure rather than intrinsic hazard alone [3,8]. The regulatory thresholds are derived based on long-term, 70-year exposure scenarios—quantified as No Significant Risk Levels (NSRLs) for carcinogenic compounds (capping excess cancer risk at 1 in 100,000) and Maximum Allowable Dose Levels (MADLs) for reproductive toxicants—the framework relies on exposure-based safe harbor levels and informational warnings rather than chemical bans [3,8], reflecting established dose–response evaluation frameworks for extrapolating quantitative safety thresholds to human exposure scenarios [9]. To achieve regulatory compliance, chemical and product manufacturers generally navigate three distinct corporate strategies: (i) accepting the chemical’s presence and applying an explicit warning label; (ii) utilizing quantitative risk assessments to prove exposure remains beneath the safe harbor thresholds, thereby avoiding the need for a warning label on the product; or (iii) enforcing a preventive ban to completely eliminate the ingredient from the portfolio [2,3,5]. However, within consumer sectors such as the cosmetic and fragrance industries, this regulatory dynamic creates market challenges due to commercial concerns regarding warning labels. Empirical findings reveal that a Proposition 65 warning significantly reduces a product’s market attractiveness and consumer purchase intent. Furthermore, consumers not only reject the flagged product but also project negative evaluations onto the retail establishments choosing to distribute them [5]. Most consumers cannot easily distinguish an intrinsic hazard from an actual risk under normal conditions of use; the market perceives these informational warnings as a sign of rejection and a confession of danger, potentially impacting brand perception [4,5].

Adding to these commercial challenges, Proposition 65 shifts the burden of proof, requiring defending companies to prove their products are safe. Driven by private enforcement provisions, the threat of litigation often overshadows science-based risk assessment. This vulnerability is exacerbated because OEHHA has not established official safe harbor levels for all listed substances, and the cost of assessing aggregate exposure can be prohibitive. Consequently, a hazard-based ban has emerged as a compliance strategy, with approximately 78% of large corporations using Proposition 65 to exclude targeted ingredients from their global supply chains via Restrictive Substance Lists (RSLs) [3]. This approach can limit ingredient availability and formulation flexibility [2]. More critically, this approach often triggers ‘regrettable substitution’—replacing a thoroughly studied, listed compound with an understudied structural analog, potentially introducing unforeseen public health risks [3,10]. To achieve regulatory compliance without compromising safety or relying on animal testing, modern regulatory science demands a shift toward New Approach Methodologies (NAMs), leveraging *in vitro* assays and computational (*in silico*) tools to provide risk characterization [2,11]. The application of quantitative structure–activity relationships (QSARs) and molecular descriptors to predict biological response thresholds builds upon foundational modeling frameworks established for volatile and fragrance constituents [12].

An example of this regulatory vulnerability manifests within the phenylpropene (alkenylbenzene) chemical family, a class of compounds that serve as natural components of high-value essential oils widely used as fragrance ingredients in cosmetic products [5,7]. Within this category, safrole represents a well-characterized carcinogen subject to a strictly enforced Proposition 65 NSRL safe harbor level of 3 µg/day [6]. In contrast, its structural analogs, estragole and methyleugenol, are highly prevalent in cosmetic fragrances but lack safe harbor thresholds, exposing manufacturers to regulatory uncertainty and forcing them to substitute ingredients that may be safe at specific concentrations [3].

**Figure 1:**
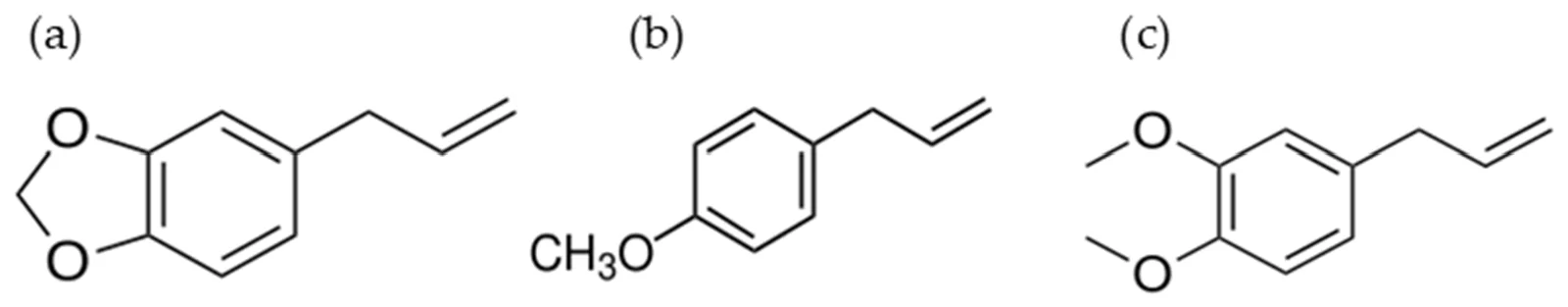
Chemical structures of the compounds studied: (**a**) safrole; (**b**) estragole; (**c**) methyleugenol.

Safrole, estragole, and methyleugenol (Figure 1) are known pro-carcinogens that are inert as parent compounds and require hepatic bioactivation to exert genotoxic and carcinogenic effects [13,14]. In the human body, this bioactivation pathway follows a two-step process: Phase I metabolism is mediated by hepatic Cytochrome P450 enzymes, predominantly the CYP1A2 isoform, which catalyzes the α-hydroxylation of the terminal allyl side chain, generating a reactive 1′-hydroxy intermediate [15]. Subsequently, Phase II conjugation occurs via sulfotransferases (SULTs), which convert this metabolite into an unstable sulfate ester [14,16]. The spontaneous dissociation of this sulfate group releases an electrophilic carbonium ion capable of binding covalently to cellular macromolecules, leading to the formation of pro-mutagenic N^2^-(deoxyguanosin-8-yl) DNA adducts [14,15]. Although competing detoxification pathways exist, such as ring *O*-demethylation or side-chain epoxidation, this shared bioactivation mechanism underpins their uniform mammalian clastogenic potential, and it provides the biological plausibility required for group-based predictive assessments [13,14].

Therefore, the aim of this study was to establish a safe harbor level (NSRL) for estragole and methyleugenol. To achieve this, we applied an *in silico* workflow based on a mechanistic read-across from safrole, integrating multiple computational lines of evidence to support regulatory compliance without animal testing [2,11].

## 2. Materials and Methods

This research quantified the topological and structural similarity within the phenylpropene category using PubChem-derived Tanimoto structural coefficients (*Tc*), followed by toxicokinetic profiling via SwissADME and an *in silico* workflow integrating ToxTree, Danish QSAR, and VEGA QSAR platforms to cross-validate genotoxic mechanisms (such as Ames mutagenicity versus mammalian micronucleus) and final carcinogenic outcomes. Furthermore, structural molecular docking simulations were conducted to evaluate the physical binding affinity, interaction patterns, and spatial proximity of safrole, estragole, and methyleugenol within the active site of the human Cytochrome P450 1A2 (CYP1A2) enzyme, which acts as the primary driver of hepatic bioactivation.

### 2.1. Topological, Physicochemical, and Pharmacokinetic Similarity Assessment

The initial phase of the read-across framework required a quantitative evaluation of the structural, physicochemical, and pharmacokinetic relationships within the phenylpropene category. The workflow design was structured in alignment with the 10-step best practice guidance framework for cosmetic ingredients established by the International Collaboration on Cosmetics Safety (ICCS) [17], in accordance with OECD Guidance Document No. 418 and ECHA Read-Across Assessment Framework (RAAF) principles [18,19]. Category qualification was established based on four predefined criteria: (1) a shared structural core (benzene ring with a terminal allyl side chain), (2) functional group homology (oxygenated aromatic ring substitutions), (3) a common bioactivation pathway (hepatic CYP-mediated α-hydroxylation), and (4) similar physicochemical descriptors. Safrole was designated as the source compound based on its established regulatory thresholds under California Proposition 65 and its complete empirical toxicological dataset, which includes in vivo carcinogenicity bioassays, quantified kinetic parameters for Phase I (α-hydroxylation) and Phase II (sulfonation) metabolism, and characterized N^2^-(deoxyguanosin-8-yl) DNA adduct formation rates. Estragole and methyleugenol were defined as target compounds. To minimize read-across uncertainty, the category boundary was defined to evaluate metabolic divergence, especially accounting for competing detoxification routes (*O*-demethylation) alongside bioactivation mechanisms across target analogs.

Two-dimensional (2D) chemical structures and canonical Simplified Molecular Input Line Entry System (SMILES) strings were retrieved from the National Center for Biotechnology Information (NCBI) PubChem database (Table 1). Structural similarity was quantified using *Tc*, calculated based on PubChem’s 881-bit 2D structure fingerprints. The bit-string comparison measures the proportion of shared chemical fragments relative to the total number of unique attributes present in both molecules, according to the following equation [20,21]:

(1) $$T_{c}\;=\;\frac{N_{AB}}{N_{A}+\;N_{B}-\;N_{AB}}$$

where

- N_AB_ represents the number of synchronized structural bits concurrently present in both compared compounds;
- N_A_ is the total number of bits set in molecule A (source compound);
- N_B_ is the total number of bits set in molecule B (target compound).

A *Tc* threshold ≥ 0.80 was utilized as the structural benchmark to validate the chemical homogeneity and clustering of the category. This threshold is a standard benchmark in chemometrics and computational toxicology, indicating a high probability that the molecules share similar biological activity and interactions with identical molecular targets [22,23].

To complement this structural assessment and validate the exposure pathways under cosmetic application conditions, an *in silico* pharmacokinetic screening was performed using the SwissADME web server-Swiss Institute of Bioinformatics, http://www.swissadme.ch (accessed on 10 June 2026) [24]. Domain suitability for SwissADME evaluations was confirmed using Bioavailability Radar ranges and BOILED-Egg model boundaries (WLOGP vs. TPSA descriptor space), ensuring target molecules fell within optimal property spaces.

The canonical SMILES strings (Table 1) were submitted to model key absorption, distribution, metabolism, and excretion (ADME) properties. This evaluation focused on parameters essential for cutaneous safety assessments, including the following:

- The consensus octanol-water partition coefficient (log Po/w) to assess lipophilicity;
- Gastrointestinal (GI) absorption;
- The skin permeation coefficient (log *Kp*, in cm/s) to quantify dermal absorption rates;
- Medicinal chemistry structural alerts (specifically the Brenk filter) to screen for potentially reactive or toxicophoric fragments.

### 2.2. Multi-Platform Computational Predictive Modeling

To construct a weight-of-evidence framework evaluating toxicological mechanisms and final endpoints, three independent *in silico* platforms encompassing both expert rule-based systems and quantitative structure–activity relationship (QSAR) models were deployed. This consensus approach balanced inherent algorithmic biases and database-specific limitations, satisfying global regulatory standards for predictive computational toxicology. The CAS numbers and canonical SMILES strings (Table 1) for the source compound (safrole) and both target compounds (estragole and methyleugenol) were evaluated across the following software suites [25,26].

To evaluate prediction reliability, model outputs were assessed against their respective Applicability Domain (AD) boundaries. VEGA predictions were evaluated using the Applicability Domain Index (ADI, scale 0–1), which combines structural similarity to training sets, descriptor ranges, fragment representation, and nearest-neighbor accuracy. Scores close to 1 indicate high structural similarity and predictive confidence, while lower values flag borderline or out-of-domain algorithmic uncertainties. Only results within the models’ applicability domain and with an ADI ≥ 0.70 were used. Predictions from the Danish QSAR Database were preferentially derived from consensus battery models combining Leadscope, SciQSAR, and CASE Ultra algorithms, considering only results within the applicability domain. Unlike statistical QSAR models, rule-based systems like ToxTree relied on decision trees and structural alerts rather than descriptor spaces; domain applicability for ToxTree was therefore confirmed when target molecular structures aligned with defined decision-tree rules.

#### 2.2.1. ToxTree

As an expert rule-based system, ToxTree—https://apps.ideaconsult.net/data/ui/toxtree (accessed on 10 June 2026)—was utilized to map qualitative mechanistic alerts from the chemical structures. The assessment was based on three rulebases:

- Revised Cramer decision tree: used to determine the broad threshold of toxicological concern (TTC) based on systemic structural features, assigning the source and target compounds into Class I (Low), Class II (Intermediate), or Class III (High) potential toxicity.
- Benigni/Bossa rulebase for carcinogenicity and mutagenicity: applied to screen for structural alerts associated with genotoxic carcinogens (specifically tracking the alkenylbenzene alert, SA38_gen) and nongenotoxic pathways (SA54_nogen), alongside automated profiling for SN1 and Michael Acceptor DNA-binding mechanisms [27].
- *In vitro* mutagenicity (Ames Test) alerts by ISS: developed by the Istituto Superiore di Sanità, this rulebase was run to screen for specific structural markers of bacterial reverse mutation (SA38_Ames).

#### 2.2.2. Danish QSAR Database

Compounds were evaluated using the Danish QSAR Database—Danish Environmental Protection Agency; https://qsardb.food.dtu.dk/db/index.html (accessed on 10 June 2026)—to obtain predictions for three endpoints:

- Genotoxicity structural alerts: The Ashby–Tennant rulebase was utilized to map potential DNA reactivity.
- Bacterial reverse mutation test (Ames Test): Both the individual *in vitro* reverse mutation model (Ames Test in *S. typhimurium*) and the Ames Test Consensus model were evaluated.
- Mammalian genotoxicity: chromosomal damage predictions were extracted via the in vitro micronucleus assay (Micronucleus v1.0.1), which was modeled through the statistical VERMEER algorithm.

#### 2.2.3. VEGA QSAR Platform

The VEGA platform—https://www.vegahub.eu/portfolio-item/vega-qsar/ (version 1.2.0, accessed on 10 June 2026)—was used to predict toxicological endpoints using statistical and fragment-based models. The following models were executed:

- Mutagenicity: The mutagenicity (Ames test) Consensus model (version 1.0.4) was applied.
- Chromosomal damage: Three independent models were run to evaluate structural clastogenicity, including the Chromosomal aberration model (Coral) (version 1.0.1), the *In vitro* micronucleus activity (IRFMN-VERMEER) (version 1.0.1), and the *In vitro* micronucleus activity (IRFMN) (version 1.0.2).
- Carcinogenicity: Four independent endpoint models was executed, namely the carcinogenicity model (CAESAR) (version 2.1.10), the carcinogenicity model (ISS) (version 1.0.3), the carcinogenicity model (IRFMN-ISSCAN-CGX) (version 1.0.1), and the carcinogenicity model (IRFMN-Antares) (version 1.0.1).

### 2.3. Molecular Docking Simulations with CYP1A2

#### 2.3.1. Protein and Ligand Preparation

Human Cytochrome P450 1A2 (CYP1A2) was selected as the primary biological target for molecular docking simulations. Although other isoforms (such as CYP2E1, CYP2C9, and CYP2C19) can contribute to alkenylbenzene oxidation at high, saturating concentrations, kinetic studies in human liver microsomes and recombinant enzymes demonstrate that CYP1A2 is the primary isoform catalyzing 1′-hydroxylation at low, physiologically relevant exposure levels [28,29]. Since consumer exposure to fragrance ingredients occurs at low levels, CYP1A2 serves as the rate-limiting enzyme in this bioactivation pathway.

The molecular docking simulation was performed using AutoDock Vina (v.1.1.2) [30]. The three-dimensional crystal structure of human CYP1A2, co-crystallized with its endogenous ligand α-naphthoflavone (ANF, also referred to as BHF), was retrieved from the Protein Data Bank (PDB ID: 2H14, resolution: 1.95 Å) [31]. The protein structure was prepared by removing the co-crystallized ligand and all water molecules, adding necessary hydrogen atoms, and assigning Gasteiger charges. The binding pocket was defined using a 30 × 30 × 30 grid box centered on the reference ligand BHF (X: 3.796; Y: 18.276; Z: 21.260).

Target compounds estragole (CID 8815) and methyleugenol (CID 7127), along with the source compound, safrole (CID 5144), were retrieved from the National Center for Biotechnology Information (NCBI) PubChem database in 3D SDF format. Structures were optimized, energy-minimized, and saved in PDBQT format, maintaining rotatable bonds as flexible during docking runs.

#### 2.3.2. Docking Execution and Protocol Validation

Docking simulations were run in triplicate for each compound at an exhaustiveness of 8 to ensure reproducible binding poses [30,32].

To validate this molecular docking workflow, a redocking procedure was executed to reproduce the experimental binding pose of BHF. The spatial discrepancy between the docked poses and the crystallographic reference was quantified by calculating the symmetry-corrected root-mean-square deviation (RMSD) over all non-hydrogen (heavy) atoms using the spyrmsd library. To preserve the exact physical coordinates within the active site, these calculations were executed in situ without structural realignment or minimization, according to the following equation:

(2) $$RMSD\;=\;\sqrt{\frac{1}{N}\sum_{i=1}^{N}{\left|\left|r_{i}^{pose}-\;r_{i}^{ref}\right|\right|}^{2}}$$

where

- N represents the total number of heavy atoms;
- r^pose^ and r^ref^ denote the Cartesian coordinate vectors of atom *i* in the docked pose and experimental reference, respectively.

A symmetry-corrected RMSD threshold of ≤2.0 Å was established for protocol validation [31,33]. Validation was achieved as the redocked BHF pose yielded an RMSD of 0.273 Å relative to the crystal structure.

#### 2.3.3. Post-Docking Spatial Overlap and Alignment Metrics

For compounds chemically distinct from the co-crystallized ligand (BHF)—where standard RMSD is topologically invalid—spatial overlap and active-site convergence were evaluated using complementary metrics [31,32]. Active site contact residues (within 5 Å of any ligand heavy atom) were extracted using MDAnalysis. Contact profile similarity between the docked pose (R_pose_) and the reference ligand (R_ref_) was calculated using the Jaccard similarity coefficient (J_contacts_) [31]:

(3) $$J\left(R_{pose},\;R_{ref}\right)=\frac{\left|R_{pose}\cap \;R_{ref}\right|}{\left|R_{pose}\cup \;R_{ref}\right|}$$

where

- R_pose_ and R_ref_ denote the sets of active site amino acid residues contacting the docked pose and experimental reference ligand, respectively;
- ∪ and ∩ represent the intersection and union operators of these contact residue sets, respectively.

Centroid distance (d_centroid_) was calculated as the Euclidean distance between geometric centers, with values $ < 2.0$ Å indicating binding pocket accommodation [33]. Core orientation consistency was evaluated via maximum common substructure RMSD (MCS-RMSD) over the shared chemical scaffold using RDKit. Finally, proximity to the catalytic site was determined by measuring minimum Euclidean and centroid-to-atom distances to the central iron (Fe) atom of the HEME cofactor [32,34].

## 3. Results and Discussion

### 3.1. Structural Topology, Physicochemical, and Toxicokinetic Profiling for Read-Across Validation

Under international regulatory frameworks (e.g., OECD and ECHA) [25,26], structural similarity alone is insufficient for read-across validation; source and target compounds must also exhibit comparable physicochemical profiles to support similar toxicokinetic behaviors. Therefore, molecular identifiers, structural parameters, and PubChem-derived Tanimoto similarity coefficients (*Tc*) are compiled in Table 2. Beyond topological and kinetic similarities, the selection of safrole as the source compound is supported by its rodent carcinogenicity potency (TD_50_) and metabolic dataset relative to target analogues. This approach aligns with OECD and ECHA read-across frameworks, establishing a conservative baseline for safety assessment.

Data in Table 2 show consistent physicochemical and topological properties across the evaluated group, supporting the ECHA RAAF criteria for category grouping [18,35]. All three compounds are low-molecular-weight alkenylbenzenes (148.20 to 178.23 g/mol). According to the SCCS Notes of Guidance, a molecular weight well below 500 Da favors passive diffusion across the stratum corneum [11]. The octanol-water partition coefficients (log *P*) align with this toxicokinetic profile, with values ranging from 2.60 to 3.40. Within regulatory frameworks, a log *P* between 1 and 4 indicates lipophilicity favorable for cutaneous absorption and systemic bioavailability, suggesting that estragole and methyleugenol exhibit comparable passive skin-permeation kinetics in cosmetic formulations relative to safrole [11,19].

Topological polar surface area (TPSA) values ranged from 9.23 to 18.50 Å^2^, with no hydrogen bond donors present. These parameters reflect lipophilic structures with low polar surface area, consistent with similar cellular distribution and potential for hepatic bioactivation [36]. *In silico* ADME profiling via SwissADME was conducted to evaluate toxicokinetic parameters [11,19]. Calculated skin permeation coefficients (log *Kp*) ranged from −4.81 cm/s for estragole to −5.60 cm/s for methyleugenol, with safrole at −5.19 cm/s. These results indicate moderate-to-high cutaneous absorption potential, suggesting that all three compounds share similar passive skin-permeation trends [11].

While all compounds showed high gastrointestinal (GI) absorption, their lower dermal penetration rates (log *Kp*) contrast with the rapid oral exposures evaluated in rodent bioassays [11]. This kinetic divergence suggests that applying oral-derived Proposition 65 NSRL thresholds to cosmetic dermal exposure represents a conservative safety approach [3,8]. Medicinal chemistry profiling identified a single Brenk structural alert for all three substances: an isolated alkene [27]. This feature corresponds to the terminal allyl side chain, aligning with molecular docking simulations where this group was positioned in catalytic proximity to the CYP1A2 HEME iron [14,15,37,38,39].

Computed *Tc* met the established threshold for chemical category clustering (*Tc* ≥ 0.80), a standard benchmark in computational toxicology for assuming shared biological activity [22,23]. The structural similarity of estragole (*Tc* = 0.82) and methyleugenol (*Tc* = 0.85) relative to safrole indicates that the open-chain methoxy groups (-OCH_3_) in the target compounds do not substantially alter the molecular topology defined by safrole’s rigid 1,3-benzodioxole ring [7]. The highest structural similarity was observed between the two target compounds (*Tc* = 0.93). Together, the physicochemical and structural parameters in Table 2 provide a supported baseline under regulatory guidelines, consistent with the principle that structurally similar compounds with comparable physical properties exhibit similar biological profiles [3,18,19].

### 3.2. Multi-Platform Computational Predictive Modeling: Mechanistic Divergence and Regulatory Implications

The integration of data from ToxTree, the Danish QSAR Database, and the VEGA QSAR generated a multi-parametric consensus toxicological profile. Only computational predictions falling within the defined applicability domains of the respective algorithms were considered to reduce prediction uncertainty. The predictive matrix is summarized in Table 3.

The multi-platform profiling highlighted both strengths and limitations of fragment-based computational toxicology [27,40]. Results across all three platforms were consistent regarding genotoxicity and mutagenicity endpoints. In bacterial systems, the integrated consensus models of both the Danish QSAR and VEGA platforms predicted a negative outcome for the Ames test across safrole, estragole, and methyleugenol. This trend aligns with established *in vitro* literature indicating that parent phenylpropenes are not direct-acting bacterial mutagens due to the absence of intrinsic electrophilic centers [27,39]. However, when evaluating mammalian chromosomal damage, the platforms showed positive predictions for the *in vitro* micronucleus assay (via the VERMEER algorithms in both Danish and VEGA suites), reflecting the clastogenic potential of this chemical class following metabolic activation [13,15].

Within VEGA’s carcinogenicity models, an algorithmic divergence was observed. The CAESAR 2.1.10 model generated a positive carcinogenicity prediction for the source compound safrole but yielded negative outcomes for target compounds estragole and methyleugenol. This divergence represents an artifact in models relying heavily on rigid structural fragment matching [27]. The CAESAR algorithm is sensitive to the intact 1,3-benzodioxole ring system unique to safrole, flagging it as a carcinogenicity alert. In contrast, estragole and methyleugenol contain open methoxy groups (-OCH_3_), which trigger automated screening branches associated with metabolic *O*-demethylation—a known detoxification pathway [39]. To resolve these discrepancies across platforms, a weight-of-evidence (WoE) evaluation was applied, prioritizing predictions based on mechanistic relevance and applicability domain. While CAESAR over-weighted *O*-demethylation, expert rule-based models (ToxTree, ISS, and ISSCAN-CGX) recognized that regardless of aromatic ring substitution, all three compounds retain the terminal allyl side chain required for Cytochrome P450-mediated α-hydroxylation. Decision-making therefore prioritized models that evaluate this shared bioactivation pathway within their defined applicability domains.

In addition to Phase I α-hydroxylation, these expert systems (ISS 1.0.3 and IRFMN-ISSCAN-CGX 1.0.1) account for Phase II biotransformation [27]. They evaluate the sulfonation catalyzed by sulfotransferases (SULTs), which converts the 1′-hydroxy metabolite into a short-lived electrophilic ester capable of forming covalent DNA adducts [13,15]. Recognizing that all three compounds undergo this shared bioactivation pathway regardless of whether the aromatic ring contains a closed methylenedioxy structure or open methoxy groups, the ISS and ISSCAN-CGX models converged on positive carcinogenicity predictions, supporting the read-across category.

From a regulatory perspective, these findings inform compliance under California Proposition 65 [3]. Concerns over potential litigation can lead consumer cosmetic brands to implement defensive, hazard-based ingredient bans [3,5]. This strategy can result in “regrettable substitutions,” where characterized components are replaced by less studied alternatives that may present unquantified risks [2,10]. Because safrole consistently generated positive predictions across valid carcinogenicity and mechanistic models, it serves as a conservative toxicological benchmark within this phenylpropene category. The target compounds, estragole and methyleugenol, share this metabolic activation pathway but exhibit attenuated hazard potential due to competing detoxification routes [15,39].

To address uncertainties in the read-across, a qualitative uncertainty evaluation was applied following OECD/ECHA guidelines [18,19]. While all category members share a rate-limiting bioactivation route—α-hydroxylation of the terminal allyl side chain yielding electrophilic 1′-sulfooxy intermediates—metabolic divergence occurs through competing detoxification pathways. In target compounds (estragole and methyleugenol), open methoxy groups (-OCH_3_) undergo efficient CYP-mediated *O*-demethylation to form readily excretable phenolic derivatives. In contrast, safrole’s closed 1,3-benzodioxole ring lacks this rapid demethylation clearance mechanism [15,28,29]. Consequently, assuming toxicological equivalence between safrole and target analogs incorporates a built-in safety margin, as competing *O*-demethylation in vivo attenuates the net metabolic fraction undergoing bioactivation in target compounds.

### 3.3. Molecular Docking Hierarchy and Mode of Action Plausibility

Molecular docking simulations against CYP1A2 (Table 4) provided mechanistic insight supporting safrole as a conservative toxicological benchmark for this phenylpropene category, aligned with OECD guidelines on category justification [19]. The observed binding affinity ranking (safrole > methyleugenol > estragole) correlates with the compounds’ established toxicological modes of action [14]. Safrole exhibited the highest binding affinity (−8.026 kcal/mol), likely due to its rigid 1,3-benzodioxole ring system. This structure forms two hydrogen bonds with active-site residues THR124 and ASP313 (Table 4), contributing to substrate stabilization within the CYP1A2 cavity [32,37]. In contrast, the lower calculated affinities of methyleugenol (−7.669 kcal/mol) and estragole (−7.443 kcal/mol) may be attributed to the flexibility of their open-chain methoxy (-OCH_3_) groups. These conformations reduce stabilizing polar interactions, yielding one hydrogen bond for methyleugenol and none for estragole [32,37].

**Figure 2:**
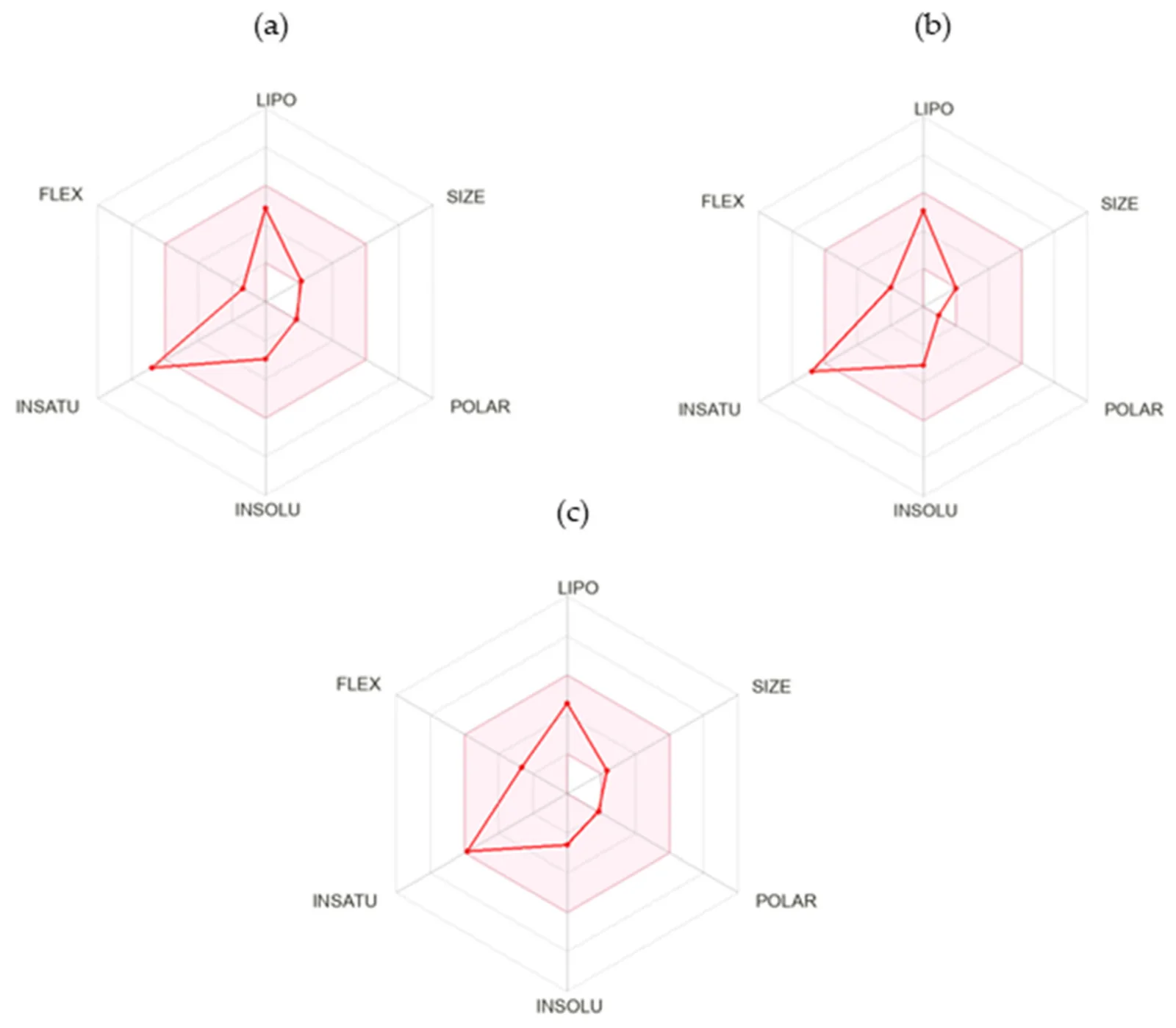
SwissADME bioavailability radars for (**a**) safrole, (**b**) estragole, and (**c**) methyleugenol. The pink-shaded area represents the optimal physicochemical space for oral bioavailability, and the red line indicates each compound’s computed profile. The overlapping profiles illustrate physicochemical consistency across the category, supporting the read-across approach. The slight deviation outside the optimal zone for unsaturation (INSATU) across all three compounds is attributed to their shared aromatic ring and terminal allyl group.

Safrole also achieved the closest catalytic proximity to the Cytochrome HEME iron (Table 4), positioning its reactive terminal allyl side chain at a distance of 7.47 Å (Figure 2). Given that P450-mediated α-hydroxylation of this specific side chain is a rate-limiting step for electrophile generation, proximity to the HEME iron in P450 docking is often associated with metabolic susceptibility [32,34]. Thus, molecular docking simulations suggested that safrole binds with optimal stability while replicating the spatial orientation and chemical interactions of the crystallographic reference (BHF), a conformation crucial for downstream catalytic activation [32], supporting the extrapolation of these interaction parameters across category members.

While molecular docking demonstrated high binding affinity between safrole and the CYP1A2 active site, these static energy evaluations do not directly determine catalytic efficiency (*kcat*/*Km*) or metabolic turnover rate. Binding affinity predictions serve as structural indicators of active-site occupancy rather than measurements of bioactivation kinetics. Liver microsomal data confirm that CYP1A2 exhibits high affinity (low Km) for 1′-hydroxylation at low exposure levels [15,29]. Nevertheless, accounting for the inherent uncertainties of docking inferences, these active-site interactions support the use of safrole as a conservative toxicological benchmark within this category.

**Figure 3:**
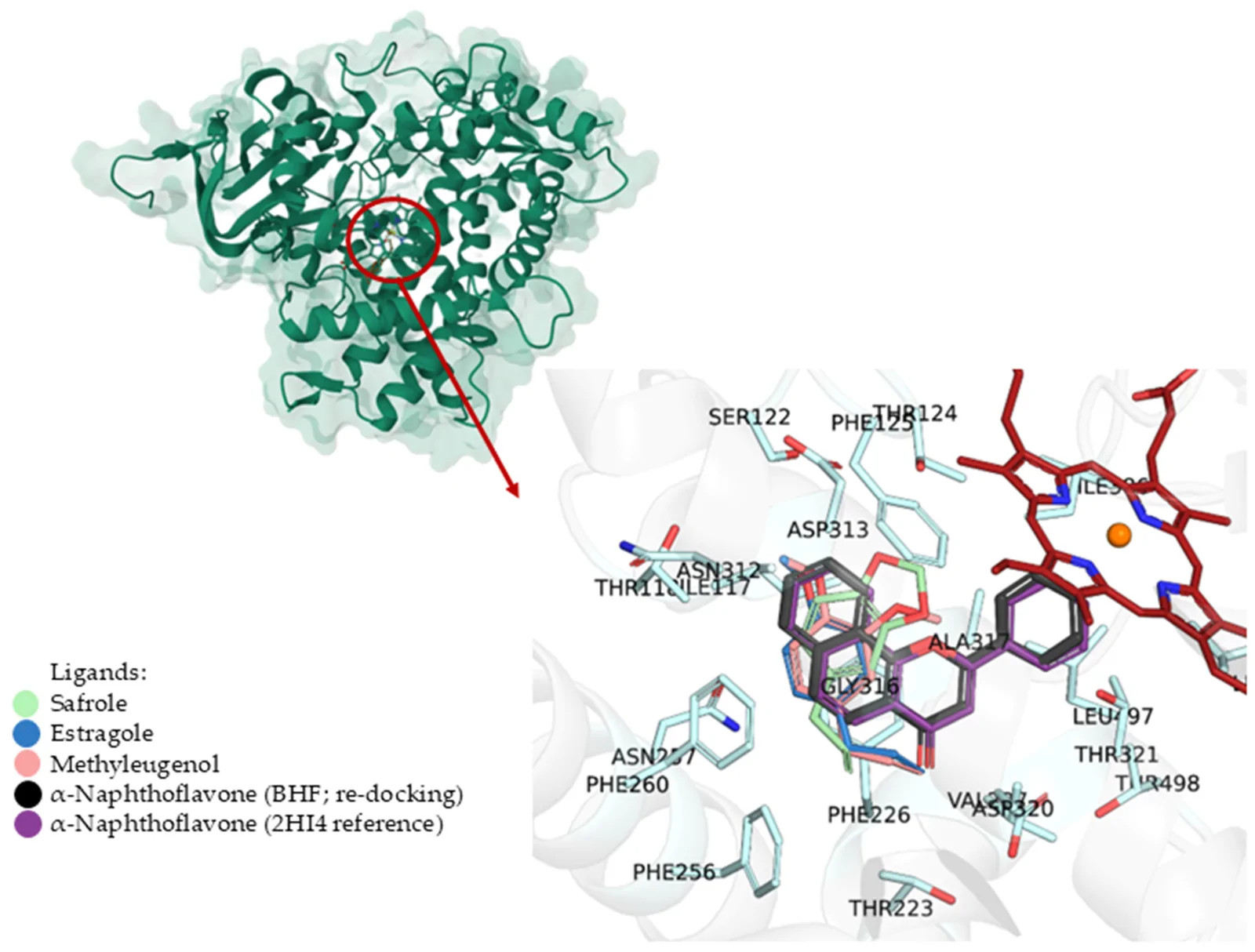
Molecular docking poses of safrole, estragole, and methyleugenol within the human CYP1A2 active site (PDB ID: 2H14). The visualization illustrates the orientation of the compounds relative to the catalytic HEME iron compared to the reference ligand, α-naphthoflavone (BHF).

The predicted binding poses of safrole, estragole, and methyleugenol within the CYP1A2 active site (PDB ID: 2H14) are shown in Figure 3, illustrating their spatial orientation relative to the catalytic HEME iron and the co-crystallized reference ligand, α-naphthoflavone. Together, these findings support safrole as a conservative toxicological reference for this phenylpropene category [34]. Consequently, applying safrole’s California Proposition 65 NSRL threshold (3 µg/day) to estragole and methyleugenol represents a precautionary risk-assessment approach for cosmetic formulations, consistent with regulatory guidelines for non-animal testing strategies [2,3,6,19].

## 4. Conclusions

This study demonstrates a non-animal Next-Generation Risk Assessment (NGRA) workflow to support safe harbor levels for estragole and methyleugenol under California Proposition 65. The high structural similarity (*Tc* ≥ 0.82) and consistent physicochemical properties support category grouping under OECD Guidance Document (No. 418) and ECHA guidelines, while multi-platform *in silico* consensus predictions addressed limitations in statistical fragment-based algorithms regarding their shared bioactivation pathway. Molecular docking with human CYP1A2 provided binding and spatial parameters supporting safrole as a conservative toxicological benchmark within the category, given its calculated binding affinity (−8.026 kcal/mol) and proximity to the HEME iron relative to target analogues.

Consequently, applying safrole’s established 3 µg/day NSRL to estragole and methyleugenol provides a quantitative threshold to evaluate consumer exposure, representing a precautionary risk-management strategy that protects public health while avoiding unnecessary ingredient substitutions in cosmetic formulations. The safe harbor thresholds established in this study define hazard limits for target analogs. Finished cosmetic product compliance calculations represent a secondary, product-specific evaluation step.

## Figures and Tables

**Table 1:** Chemical identifiers, registration numbers, and canonical SMILES strings for the evaluated phenylpropenes.

Compounds	PubChem CID	CAS Number	SMILES
Safrole	5144	94-59-7	C=CCC1=CC2=C(C=C1)OCO2
Estragole	8815	140-67-0	COC1=CC=C(C=C1)CC=C
Methyleugenol	7127	93-15-2	COC1=C(C=C(C=C1)CC=C)OC

CID: Compound Identifier; CAS Number: Chemical Abstracts Service Number; SMILES: Simplified Molecular Input Line Entry System.

**Table 2:** Integrated molecular identifiers, core physicochemical properties, and Tanimoto similarity coefficients (*Tc*) for the evaluated alkenylbenzenes.

Parameters	Safrole (Source)	Estragole (Target 1)	Methyleugenol (Target 2)
Molecular formula	C_10_H_10_O_2_	C_10_H_12_O	C_11_H_14_O_2_
Molecular weight (g/mol)	162.19	148.20	178.23
Octanol-water partition coefficient (log *P*)	2.60	3.40	3.00
Topological polar surface area (TPSA) (Å^2^)	18.50	9.23	18.50
Hydrogen bond donor count	0	0	0
Hydrogen bond acceptor count	2	1	2
Consensus log P_o/w_	2.53	2.86	2.56
Log *Kp* (Skin Permeation, cm/s)	−5.19	−4.81	−5.60
GI absorption	High	High	High
Brenk structural alerts	1 (isolated_alkene)	1 (isolated_alkene)	1 (isolated_alkene)
Tanimoto Coefficient (*Tc* vs. safrole)	1.00	0.82	0.85

**Table 3:** Consensus toxicological predictions for safrole, estragole, and methyleugenol across *in silico* platforms.

Predictive Endpoint	Safrole (Source)	Estragole (Target)	Methyleugenol (Target)
1. ToxTree (v3.1.0)			
Revised Cramer decision tree	High (Class III)	High (Class III)	Low (Class I)
Benigni/Bossa carcinogenicity alert	Positive (SA38)	Positive (SA38)	Positive (SA38)
DNA binding mechanism	Positive (SN1/Michael)	Positive (SN1/Michael)	Positive (SN1/Michael)
*In vitro* mutagenicity (Ames ISS)	Positive (SA38_Ames)	Positive (SA38_Ames)	Positive (SA38_Ames)
2. Danish QSAR Database			
Ashby structural alerts (DNA Reactivity)	Positive	Positive	Positive
Bacterial reverse mutation (Ames Test)	Negative	Negative	Negative
Ames test consensus model	Negative	Negative	Negative
Micronucleus assay (VERMEER)	Positive	Positive	Positive
3. VEGA QSAR Platform			
Mutagenicity (Ames) consensus 1.0.4	Negative	Negative	Negative
*In vitro* micronucleus (IRFMN-VERMEER)	Positive	Positive	Positive
*In vitro* micronucleus (IRFMN 1.0.2)	Negative	Negative	Negative
Chromosomal aberration (Coral 1.0.1)	Out of domain	Out of domain	Negative
Carcinogenicity (CAESAR) 2.1.10	Positive	Negative	Negative
Carcinogenicity (ISS) 1.0.3	Positive	Positive	Positive
Carcinogenicity (IRFMN-ISSCAN-CGX)	Positive	Positive	Positive
Carcinogenicity (IRFMN-Antares)	Positive	Out of domain	Positive

ISS: Istituto Superiore di Sanità.

**Table 4:** Molecular docking parameters and active-site interactions with human CYP1A2.

Compound	Binding Affinity (kcal/mol)	Inhibition Constant (Ki)	Ligand Efficiency (LE)	Centroid Dist. (Å)	MCS-RMSD (Å)	Jaccard Index	Min. Fe Dist. (Å)	Hydrogen Bonds (Å)	Hydrophobic Interactions	π-Stacking
BHF (Control)	−13.23	200.58 pM	0.630	0.24	0.27	1.00	5.03	N/A	N/A	N/A
Safrole (Source)	−8.026	1.31 µM	0.669	2.02	1.16	0.70	7.47	THR124 (3.2), ASP313 (3.3)	THR223, PHE226, PHE256, PHE260	None
Methyleugenol (Target)	−7.669	2.39 µM	0.590	2.23	0.96	0.76	7.77	ASP313 (3.9)	THR118, PHE226, PHE256, ASP320, THR223, PHE260	PHE226
Estragole (Target)	−7.443	3.50 µM	0.677	2.77	1.18	0.67	9.94	None	THR118, PHE226, PHE256, ASP320, THR223	PHE226, PHE260

BHF: endogenous reference ligand (α-naphthoflavone, PDB ID: 2H14); Ki: predicted inhibition constant; LE: ligand efficiency; Centroid Dist.: Euclidean distance between the geometric centroids of the docked ligand pose and the crystallographic reference ligand; MCS-RMSD: maximum common substructure root-mean-square deviation computed over the shared scaffold; Jaccard Index: contact residue similarity score within a 5 Å active-site boundary; Min. Fe Dist.: minimum Euclidean distance between any non-hydrogen atom of the docked ligand and the central iron (Fe) atom of the catalytic HEME group; N/A: not applicable.

## Data Availability

The original contributions presented in this study are included in the article. Further inquiries can be directed to the corresponding author.

## Abbreviations

The following abbreviations are used in this manuscript:

2D	Two-Dimensional
3D	Three-Dimensional
ADME	Absorption, Distribution, Metabolism, and Excretion
ADI	Applicability Domain Index
ANF	α-Naphthoflavone
BHF	α-Naphthoflavone (endogenous ligand identifier in PDB 2H14);
CAS	Chemical Abstracts Service
CID	Compound Identifier (PubChem)
CYP1A2	Cytochrome P450 Family 1 Subfamily A Member 2
DNA	Deoxyribonucleic Acid
ECHA	European Chemicals Agency
GI	Gastrointestinal
ISS	Istituto Superiore di Sanità
K_i_	Predicted Inhibition Constant
K_p_	Skin Permeation Coefficient
LE	Ligand Efficiency
MADL	Maximum Allowable Dose Level
MCS	Maximum Common Substructure
MCS-RMSD	Maximum Common Substructure Root-Mean-Square Deviation
NAMs	New Approach Methodologies
NCBI	National Center for Biotechnology Information
NGRA	Next-Generation Risk Assessment
NSRL	No Significant Risk Level
OECD	Organisation for Economic Co-operation and Development
OEHHA	Office of Environmental Health Hazard Assessment
PDB	Protein Data Bank
QSAR	Quantitative Structure–Activity Relationship
RAAF	Read-Across Assessment Framework
RMSD	Root-Mean-Square Deviation
RSLs	Restrictive Substance Lists
SCCS	Scientific Committee on Consumer Safety
SDF	Structure-Data File
SMILES	Simplified Molecular Input Line Entry System
SULT	Sulfotransferase
*Tc*	Tanimoto Coefficient
TPSA	Topological Polar Surface Area
TTC	Threshold of Toxicological Concern
